# A three-model comparison of the relationship between quality, satisfaction and loyalty: an empirical study of the Chinese healthcare system

**DOI:** 10.1186/1472-6963-12-436

**Published:** 2012-11-30

**Authors:** Ping Lei, Alain Jolibert

**Affiliations:** 1 Groupe Ecole Supérieure de Commerce Chambéry Savoie, France, 12 Avenue du Lac d’Annecy, Le Bourget du Lac Cedex, Chambéry, Savoie, 73381, France; 2 Centre d’études et de Recherches Appliquées à la Gestion de Université Pierre Mendes France, Grenoble 2, France

**Keywords:** Perceived quality, Patient satisfaction, Patient loyalty, Models comparison, Health service

## Abstract

**Background:**

Previous research has addressed the relationship between customer satisfaction, perceived quality and customer loyalty intentions in consumer markets. In this study, we test and compare three theoretical models of the quality–satisfaction–loyalty relationship in the Chinese healthcare system.

**Methods:**

This research focuses on hospital patients as participants in the process of healthcare procurement. Empirical data were obtained from six Chinese public hospitals in Shanghai. A total of 630 questionnaires were collected in two studies. Study 1 tested the research instruments, and Study 2 tested the three models. Confirmatory factor analysis was used to assess the scales’ construct validity by testing convergent and discriminant validity. A structural equation model (SEM) specified the distinctions between each construct. A comparison of the three theoretical models was conducted via AMOS analysis.

**Results:**

The results of the SEM demonstrate that quality and satisfaction are distinct concepts and that the first model (satisfaction mediates quality and loyalty) is the most appropriate one in the context of the Chinese healthcare environment.

**Conclusions:**

In this study, we test and compare three theoretical models of the quality–satisfaction–loyalty relationship in the Chinese healthcare system. Findings show that perceived quality improvement does not lead directly to customer loyalty. The strategy of using quality improvement to maintain patient loyalty depends on the level of patient satisfaction. This implies that the measurement of patient experiences should include topics of importance for patients’ satisfaction with health care services.

## Background

China’s healthcare system is on the brink of major reform, stimulated by a multitude of forces that are driving this change [1]. Since 2002, the Chinese government has primarily allocated its healthcare funding to graduates of medical schools and public hospitals in major cities in an effort to improve the quality of patient care. The strong government commitment to improving health services and patient satisfaction was further emphasized after the 2003 SARS outbreak, when in 2005 the “Year of Hospital Management Reform” was declared, with the key theme of “the patient comes first, improve the quality of service.” In this way, the Chinese government articulated its pledge to the healthcare sector and emphasized the need for public and private sector cooperation [1]. These actions demonstrate the government’s commitment to developing hospital management and organization in a patient-centered manner to increase patient satisfaction.

Simultaneous with the government’s new commitment, Chinese patients are becoming more knowledgeable and active in “managing” their healthcare experiences. In major cities, there are increasing numbers of individuals who are taking “responsibility” for their healthcare. There is increasing demand for improved hospital-based services, patients are becoming better informed, and, in turn, more demanding about the type and quality of health services they expect to receive. The government has, in fact, already encouraged the “voice of the consumer” by soliciting patient feedback in evaluating hospitals to help improve the level of patient care.

Because of these circumstances, Chinese hospitals are now operating in a new, complex and uncertain environment. The current transformation from a Communist system to a competitive healthcare market is obliging hospital providers to deal with decreased funding and increased competition. Facing this situation, providers must learn to cost-effectively satisfy the needs and desires of their patients. As a result, providers and policymakers are urgently seeking a clear understanding of the quality, satisfaction and loyalty intention relationship in the Chinese healthcare market.

Many researchers have studied patient satisfaction in Western healthcare services [2-7], and there are some that have studied patient satisfaction in Hong Kong and Taiwan [8-10]. However, to date no research has focused on mainland China’s healthcare market.

Over the past 30 years, a large number of consumer behavior studies have explored the links between quality, consumer satisfaction and loyalty in Western cultures, yet no consensus has been reached. Three theoretical models can, however, be found in the literature [11].

Faced with three contradictory theoretical models, it is challenging to determine which model is most appropriate to utilize in studying healthcare services in mainland China. The purpose of this study is to analyze the relationships between perceived quality, patient satisfaction and loyalty intentions in mainland China’s healthcare system.

The empirical data for this study was collected in Shanghai (eastern China), where patients have substantial freedom to choose their medical providers.

### Theoretical background and research hypotheses

Our conceptual models focus on the relationships between perceived quality, loyalty and satisfaction. Three models can be built from the existing literature (Table 1).

**Table 1:** Hypotheses and supporting literature

	**Hypotheses**	**Supporting literature**
H1:	Customer satisfaction mediates perceived quality and loyalty intention relationship	[5,6,9,13-22,27,53]
H2:	Perceived quality and customer satisfaction influence customer loyalty intention with equal weight	[11,13,18,29-32]
H3:	Perceived service quality mediates the relationship between customer satisfaction and customer loyalty intention	[11,15-17,33,34]

### Customer satisfaction mediates the quality–loyalty relationship

Perceived quality is considered the antecedent of satisfaction and customer loyalty. Therefore, customer loyalty stems primarily from perceived quality. Perceived quality directly influences customer loyalty *and* customer satisfaction. Therefore, customer satisfaction partially mediates the quality–loyalty relationship [12-14].

Several studies support this model [15-22]. Based on this approach, the process of achieving satisfaction has been described as follows. Before buying, consumers form expectations of a specific product or service. Then, consumption induces a perceived quality level that is influenced by the difference between actual quality perceptions and the expectations of quality [23,24]. If perceived quality is confirmed, then customers are satisfied. Intensity of customer loyalty is then influenced by the degree of customer satisfaction, and perceived quality is considered to influence customer loyalty.

However, the observed relationship between perceived quality and customer loyalty relies on customer satisfaction, as customer satisfaction is a mediator variable in the quality–loyalty relationship [25]. Perceived quality can be a root cause of customer loyalty. Customer satisfaction can easily be added as a third variable to the quality–loyalty relationship, wherein perceived quality causes satisfaction, and customer satisfaction causes loyalty intention. Therefore, in the mediation model, when customer satisfaction is introduced into the perceived quality–loyalty relationship, the path coefficient of this relationship drops to a non-significant level or disappears [26].

**Figure 1:**
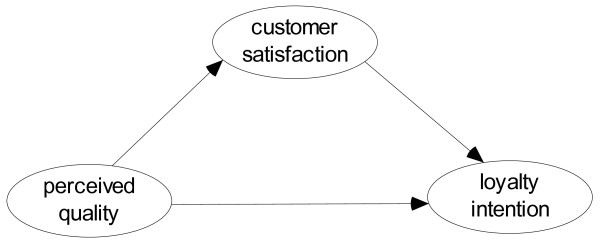
Quality–satisfaction–loyalty model.

In healthcare services, several studies have used perceived quality to assess patient satisfaction [5,8,9,13,14,27]. They indicate that patient satisfaction is the key indicator in determining the relationship between perceived healthcare quality and patient loyalty intention [8,28]. This approach considers *customer satisfaction as a mediator between perceived quality and loyalty intention* as shown in Figure 1.

### Perceived quality and customer satisfaction are one and the same

In this model, perceived quality and customer satisfaction constructs are placed at the same level [11]. Based on this approach, these two constructs have an equivalent effect on customer loyalty intention [29].

From a nomological point of view, the two concepts are separable theoretical constructs if they occupy unique positions in a nomological network as determined by unique sets of antecedent causes, consequential effects or both [30]. Conversely, if two concepts share the same theoretical antecedents and consequences, then they are “structurally equivalent” or logically isomorphic. In this case, the standard definitions of quality and satisfaction share the same antecedents (expectation and perception of the purchase experience) and consequences (both lead to loyalty intention). The positions of quality and satisfaction in this nomological network are not unique, but are structurally interchangeable [29].

**Figure 2:**
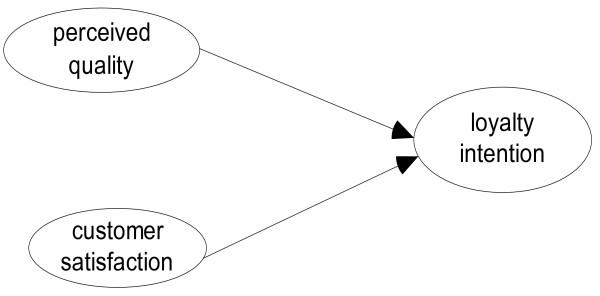
Quality and satisfaction as “one and the same” model.

Several healthcare service studies have used perceived quality as a patient satisfaction measure [13,18,31,32]. They suggest that meeting patient expectations is essential to maintaining a good patient–provider relationship [32]. However, to meet a patient’s expectations, healthcare providers must know their patients and understand their expectations. Because expectation has been defined as customer desire, service expectations do not represent what service providers offer in reality, but rather what they should offer. That is, patient satisfaction is more likely to be determined by how well provider performance fulfills innate needs, wants or desires, rather than how performance compares with presumed predictions [31]. This approach considers that *perceived quality and customer satisfaction influence customer loyalty intention with equal weight*, as represented in Figure 2.

### Perceived quality mediates the relationship between satisfaction and loyalty

According to this approach, customer satisfaction influences customer loyalty intention directly, but also influences perceived quality [11,33]. Therefore, perceived quality is a mediator between customer satisfaction and customer loyalty.

 Although there is widespread agreement that customer satisfaction is induced by performance quality, customer satisfaction may largely influence perceived quality as well. For instance, a customer might be satisfied with a particular service, but they do not think the service is of high quality. This confirms that quality evaluation is influenced by customer satisfaction, and customer satisfaction can be modeled as an antecedent of perceived quality. Using this interpretation, perceived quality is built mainly on previous experiences of (dis)satisfaction related to discrete transactional episodes [15-17]. Therefore, satisfaction is an emotional reaction that results from an intrapersonal comparison of customer expectation with the evaluation of a single product or service encounter [11]. This emotional state of satisfaction leads to an overall attitude regarding perceived quality [33,34]. In this approach, multiple satisfaction evaluations contribute to an overall perceived quality evaluation, leading to the conclusion that customer satisfaction is an antecedent of perceived quality. Perceived quality thus mediates the satisfaction–loyalty relationship [11].

**Figure 3:**
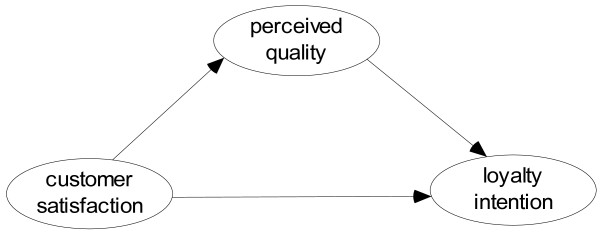
Satisfaction–quality–loyalty model.

There are studies documenting perceived quality as mediating customer satisfaction and customer loyalty in some service industries [11,33]. However, a broader review of the literature reveals a limited number of studies addressing perceived quality as mediating the satisfaction–loyalty relationship in the healthcare sector. Therefore, this research studies the relationship of quality as a mediator between satisfaction and loyalty in the Chinese healthcare sector. This approach considers *perceived service quality as a mediator of the relationship between customer satisfaction and customer loyalty intention* as shown in Figure 3.

## Methods

### Data collection

This study used inpatients as research participants to assess the relationship between perceived quality, patient satisfaction and patient loyalty intention constructs.

### Hospital sample

The six hospitals chosen for the studies were located in Shanghai, China. Based on the classification of Shanghai hospitals, we selected among the city’s 33 tertiary public hospitals. Tertiary hospitals are general hospitals located in the city, with a bed capacity exceeding 500. These hospitals treat local residents and patients with difficult and complex diseases from all over the country [35]. Among the 33 tertiary hospitals, 6 were selected for our study (Table 2). They ranged from the largest urban teaching hospitals (Fudan University Zhongshan, Huashan, Jinshan and Shanghai No. 5 People’s Hospital) to mid-sized municipal hospitals (Shanghai Oriental Hospital and Shanghai Minhang District Hospital). Among the six hospitals, two were located in urban areas, two in suburban areas and two in rural settings. The six hospitals are the largest from among the three categories of hospitals. Therefore, the selected hospitals represent the Chinese inpatient population fairly well.

**Table 2:** Sample of six public hospitals, Shanghai, 2009 and number of questionnaires

**Hospital name**	**Number of beds**	**Number of questionnaires**
Shanghai Zhongshan Hospital	1,700	150
Shanghai Huashan Hospital	1,326	130
Shanghai Dongfang Hospital	1,000	100
Shanghai Jinshan Hospital	600	60
Shanghai Minhang District Centre Hospital	800	80
Shanghai No.5 People’s Hospital	800	80

### Inpatient sample

Data were collected by four non-medical students. These students were trained to collect data on hospital patients who underwent surgery in the 30 days prior to administration of the questionnaire. Data were collected from the inpatient healthcare services department in the six public hospitals. A questionnaire was given to a systematic probability sample of individuals in two phases.

Of the 6,200 licensed beds in the 6 hospitals (Table 2), a total of 800 inpatients were selected according to a systematic sample procedure using odd numbered beds, from a bed number list established at each hospital. Questionnaires were distributed to participants who underwent surgery in the 6 hospitals in the 30 days prior to the administration of the questionnaire. A quota sampling based on the number of beds per hospital was used to obtain the number of inpatients to be questioned per hospital. Table 2 provides the number of questionnaires per hospital. Of the 800 questionnaires, 200 were distributed in the first phase, and 600 were distributed in the second phase.

With a response rate of 78 percent, 646 surveys were collected over the two phases (150 questionnaires were collected in the first phase and 496 were collected in the second phase). Empty beds and patients who were not in a position to answer constituted non-responses. Sixteen surveys were discarded because of missing data. The final sample included 630 responses (150 in the first phase and 480 in the second phase) available for statistical analysis.

The sample was made up as follows: 51 percent males and 49 percent females (aged between 20 and 65 years); 48 percent had less than a high school level education, 27 percent had a high school education, 13 percent were university graduates and 11 percent were post-graduates; 25 percent had a monthly income below $250, 57 percent between $250–$500, 8 percent between $500–$750 and 10 percent had a monthly income of more than $750. We did not consider patients under 20 years of age because of the risk of influence by staff and researchers [4] or those aged over 65 because Medicare Insurance issues related to retirement might interfere with respondent judgment [36].

Patients’ personal information was obtained from the hospital administration. To protect patient privacy and encourage the free expression of patient opinions, we did not include information such as patient name, address or diagnosis in the study. An explanatory note describing the study to respondents was placed at the beginning of the questionnaire. All participants were asked to confirm their agreement to participate before the actual survey was administered and all of them confirmed.

### Measures

The patient-perceived quality scale was developed based on the SERVQUAL instrument as recommended by Parasuraman, Zeithaml and Berry [37]. Based on its original form, SERVQUAL contains 22 pairs of reflective 7-point Likert scales (1 = strongly disagree, 7 = strongly agree). One half of these items measure the patients’ expected level of health service quality. The other half measures the perceived level of health service quality provided by hospitals. Perceived quality is measured using disconfirmation scores based on patient healthcare service quality perceptions minus service quality expectations (P–E).

Based on Cho et al. [8] and Fitzpatrick [38] patient satisfaction was measured using an overall measure of satisfaction rating, because it reflects the personal preferences of the patient, patient expectations and the reality of the care received [3]. Furthermore, it suppresses the validity and reliability bias observed when using the determinant of satisfaction [39]. Three reflective items were used: 1) How would you rate the overall quality of service provided by your hospital? 2) Thinking about the hospital overall, please rate the value you feel you get for your money. 3) Overall, how satisfied are you with your hospital? All three used 10-point semantic differential scales ranging from 1 to 10 (1 = very satisfied, 10 = very dissatisfied). Despite the existing controversy regarding their respective properties [40], 10-point scales were preferred to the usual 5-point scales because in health care satisfaction studies, they show higher validity and explanatory power than 5-point scales and the same non-response rate [41]. We used an inverted score scale to avoid response style bias, such as consistently responding yes. This choice is quite common in investigations where one can justifiably fear a “positive” bias, as is the case in China (and other cultures where individuals tend not to say no directly). According to Baumgartner and Steenkamp [42], and Peter and Churchill [43], studies show that an inverted score scale does not lower scale reliability.

Word of mouth (WOM) is used as a reflective indicator of patient loyalty. In medical services, loyalty through repeat patronization is not pertinent, whereas patient WOM [44] has an important impact on responses for several reasons [5]. First, it involves face-to-face communication between patients potentially possessing concrete information based on vivid experiences. Second, patient WOM originates from non-firm, non-marketing sources and is likely to be perceived as more credible than communications from marketers. Third, negative patient WOM can be extremely damaging because it is generally more widely communicated than positive WOM [5]. Thus, we employed WOM as a loyalty intention instrument in this study. A single item was measured using a 7-point Likert scale: “Will you recommend this hospital to someone who seeks your advice?” According to the recommendation of Bergkvist and Rossiter [45,46], “a carefully crafted single-item measure of a concrete construct is at least as valid as multiple-item measures of the same construct, and the use of a multiple-item measure then is not necessary” [46].

This study used a back translation procedure in two phases. During the first phase, the original questionnaire was translated from English into standard Mandarin Chinese by a Chinese English professor from Shanghai Foreigner Language School. In the second phase, a private translation company translated the Chinese questionnaire back into English. No differences were found between the two translations. This process has the advantage of pinpointing misinterpretations and misunderstandings before they reach the public [47]. Back translation therefore provides a test of content validity of our scales.

The questionnaire was administered in two different contexts identified as “Study 1” and “Study 2”. Study 1 tested the questionnaire developed from the literature review and the constructs of reliability and validity. Study 2 used the constructs of Study 1 and tested each of the three models (Table 3).

**Table 3:** Descriptive statistics: means and standard deviations—Study 1

**Patient-perceived service quality items**	**Statistics**	
**Mean and standard deviation**	**M**	**SD**
1 Prompt service to patients	0.82	0.99
2 Employees are consistently courteous	0.61	0.86
3 Employees deal with patients in a caring fashion	0.64	0.84
4 Providing services at the promised time	0.65	0.88
5 Employees understand the needs of patients	0.79	0.99
6 Visually appealing materials associated with the service	0.64	0.96
7 Having the patient’s best interest at heart	0.93	1.00
8 Willing to help patients	0.69	0.98
9 Maintaining error-free records	0.55	0.76
10 Keeping patients informed about when service will be performed	0.53	0.78
11 Providing service as promised	0.74	0.92
12 Employees instill confidence in patients	0.71	1.03
13 Employees have the knowledge to answer patient questions	0.75	0.99
14 Dependability in handing patients’ requests	0.66	0.89
15 Readiness to respond to patients’ requests	0.77	1.06
16 Performing services right the first time	0.70	0.96
17 Visually appealing living rooms & environments	0.95	1.08
18 Giving patients individual attention	0.91	1.07
19 Employees have a neat, professional appearance	0.43	0.74
20 Convenient business hours	0.77	0.98
21 Modern living room facilities & equipment	0.89	1.00
22 Making patients feel safe in their transactions	0.68	0.91
**Patient satisfaction items**		
How would you rate the overall quality of service provided by your hospital? (overall quality)	7.36	1.30
Thinking about this hospital overall, please rate the value you feel you get for your money (value)	7.10	1.38
Overall, how satisfied are you with this hospital? (overall satisfaction)	7.43	1.39
**Loyalty intention item**		
Recommend hospital to someone who seeks your advice	5.80	1.35

### Model estimation

To assess the construct validity of the measures prior to model estimation, a three-step data analysis was undertaken [48,49]. First, a principal component analysis using a varimax rotation was performed to eliminate cross-loading items and to optimize scale validity and reliability. Second, the validity of each measurement was evaluated by conducting confirmatory factor analysis [50]. Third, convergent validity and discriminant validity were assessed for construct validity [51]. In this research, we used perceived quality and customer satisfaction influencing customer loyalty intention as a single item [45,46].

The structural equations modeling (SEM) was used to estimate the relationships in our models. This technique was used to investigate patient satisfaction [4,52]. According to Iacobucci et al. [53], SEM performs better than regressions for mediation analysis. The software used was AMOS 5.0. Reflective measures indicators were used to build the different constructs of perceived quality, loyalty and satisfaction.

### Ethical considerations

Ethical approval was obtained from the Research and Ethics Committee of the Centre of Hospital Management at the China Shanghai Fudan Medical University. Permission was also obtained from management at each of the hospitals participating in the study. Written consent to participate in the study was obtained from all study participants. The methodology used in this study followed the principles of the Helsinki Declaration.

## Results

### Study 1: measurement reliability and validity

Study 1 analyzed the reliability and validity of the measures. The questionnaire was based on the prior literature review. Using an iterative process, we removed items with non-significant loadings or loadings on multiple factors. By doing so, we ultimately revised the items and arrived at an instrument containing items that only directly assessed the construct presented in Table 4. This process left us with a shortened scale of five items loaded on one factor for the healthcare perceived quality concept. Three items were loaded on one factor for patient satisfaction. Confirmatory factor analysis results provided strong support for each related dimensional structure (Table 5).

**Table 4:** CFA model fit for each adapted dimensional structure

**Study 1 Instrument adaptation**	**Standardized loading**	**Variance extracted**	**Construct reliability**
**Health care perceived quality (5 items)**		0.722	0.903
Providing services at the promised time	0.819		
Providing service as promised	0.846		
Employees have the knowledge to answer patient questions	0.875		
Readiness to respond to patient requests	0.860		
Performing services right the first time	0.849		
**Patient satisfaction (3 items)**		0.852	0.913
How would you rate the overall quality of service provided by your hospital?	0.932		
Thinking about this hospital overall, please rate the value you feel you get for your money.	0.905		
Overall, how satisfied are you with your hospital?	0.934		

**Table 5:** Confirmatory analysis of adapted dimensional structure CFA model fit)

**Test of constructs**	**Chi-Square (df)**	**GFI**	**AGFI**	**RMR**	**SRMR**	**RMSEA**
Perceived quality	3.507(5)	0.991	1.000	0.012	0.0141	0.000
Customer satisfaction	2.254(2)	0.990	0.999	0.079	0.0095	0.029

The overall model fit of the measurement model was good. The chi-square value was 34.90 with 25 degrees of freedom; the p-value was less than 0.05. Model fit indices were also good according to Hu and Bentler [54], goodness of fit (GFI) = 0.949, adjusted goodness of fit (AGFI) = 0.909, standardized root mean residual (SRMR) = 0.0350 and root mean square error of approximation (RMSEA) = 0.05. The average variance extracted [55] for the perceived quality construct was 0.672, and the customer satisfaction construct was equal to 0.660. In this study, the average variance extracted (AVE) was also used to evaluate discriminant validity between perceived quality and customer satisfaction constructs. According to Fornell and Larcker [55], if the AVE for each construct is greater than the squared correlation between the constructs, it confirms the discriminant validity. Examining the correlations between the perceived quality and customer satisfaction constructs, the correlation between these two constructs was 0.60, and the squared correlation was 0.36. Therefore, discriminant validity between these two constructs was checked, and the two constructs were distinct.

### Study 2: model test and comparison

In this study, we used the scale from Study 1. Perceived service quality was measured using a 5-item scale, satisfaction was measured using a 3-item scale and loyalty intentions were measured by a single-item scale. Data collection was from the same sample of hospitals as in Study 1. Six hundred questionnaires were distributed at this stage for the second study, 480 surveys were completed before the deadline, giving an 80 percent response rate. Both exploratory and confirmatory factor analyses were performed to test the shortened subscale. The hypothesized relationships between perceived quality, customer satisfaction and customer loyalty intention constructs were tested via SEM.

The overall model fit of the modified measurement model was still good. The chi-square value was 67.81 with 25 degrees of freedom, with p < 0.05, GFI = 0.970, AGFI = 0.95, SRMR = 0.0243 and RMSEA = 0.06. The AVE for the perceived quality construct was equal to 0.684, and customer satisfaction was equal to 0.717, which were greater than the squared correlation (0.399) between these two constructs. Thus, the discriminant validity of perceived quality and customer satisfaction constructs were confirmed.

After the evaluation of construct validity, these three research models of the relationship between perceived quality, patient satisfaction and patient loyalty intention constructs were tested simultaneously using SEM. The maximum likelihood method was used to investigate the covariance matrix of each item. The goodness of fit of the model was evaluated using absolute and relative indices. The value of GFI, RMSEA and SRMR were checked according to Hu and Bentler [54]. The model comparison was determined by calculating the difference in ***X***^**2**^ values [48]. The results of the model comparisons are reported in Table 6.

**Table 6:** Model results

**Model 1 Q-S-L**	***X***^**2**^	**df**	***X***^**2**^**/df**	**R**	**t**	***R***^***2***^	**TLI**	**GFI**	**AGFI**	**SRMR**	**RMSEA**
Stage 1:Q–S	59.54	19	3.13	0.630	13.38*	0.398	0.977	0.970	0.943	0.0258	0.067
S–L	6.71	2	3.35	0.481	11.07*	0.231	0.995	0.993	0.965	0.0146	0.070
Q–L	14.21	9	1.57	0.262	2.95*	0.063	0.982	0.970	0.931	0.0339	0.060
Stage 2:Q–S–L	34.90	25	1.40			0.366	0.980	0.949	0.909	0.0350	0.052
S←Q				0.605	6.998*						
L←S				0.604	5.946*						
L←Q				0.110	1.16						
**Model 2 Q=S**											
	89.03	26	3.42			0.317	0.906	0.895	0.817	0.2680	0.128
L←S				0.56	6.18*						
L←Q				0.067	0.761						
**Model 3 S–Q–L**											
Stage 1:S–Q	59.54	19	3.13	0.630	13.27*	0.398	0.977	0.970	0.943	0.0258	0.067
Q–L	14.21	9	1.57	0.262	2.95*	0.063	0.982	0.970	0.931	0.0339	0.060
S–L	6.71	2	3.35	0.481	11.07*	0.231	0.995	0.993	0.965	0.0146	0.070
Stage 2:S–Q–L	34.90	25	1.40			0.366	0.980	0.949	0.909	0.0350	0.052
Q←S				0.605	6.998*						
L←Q				0.110	1.16						
L←S				0.604	5.946*						

*Q*, perceived quality; *S*, customer satisfaction; *L*, customer loyalty intention.

* p < 0.05.

### Test of the three models

**Model 1** tests the mediation role of customer satisfaction in the relationship between perceived quality and customer loyalty intention. Using Baron and Kenny’s mediation model [26], two stages and four steps of data analysis were used during data interpretation. In stage 1, the direct links between quality–satisfaction, satisfaction–loyalty and quality–loyalty were tested (Table 6). Stage 2 introduced these three constructs into a mediation model. As shown in Table 6, the regression coefficient indicated a strong and positive effect of patient perceived quality on patient satisfaction (0.63, t = 13.38, p < 0.05). Patient satisfaction strongly and positively influenced patient loyalty intention (0.48, t = 11.07, p < 0.05). The standardized path coefficient indicated that patient-perceived quality statistically and positively influenced patient loyalty intention (0.262, t = 2.95, p < 0.05). Stage 2 tested the mediation role of customer satisfaction in the relationship between perceived quality and loyalty intention. The standard coefficients between patient perceived quality and patient loyalty intention were not statistically significant (0.11, t = 1.16, p = 0.245). This indicated that patient perceived quality and loyalty relationship depend on the level of patient satisfaction.

The overall model fit for model 1 was also acceptable, as indicated by the absolute fit and incremental fit indices (Table 6). The chi-square was 34.90 with 25 degrees of freedom, SRMR = 0.035, GFI = 0.949 and RMSEA = 0.052. The model fit indices showed that the model fits the data. Data results indicated a complete mediation role of customer satisfaction in the relationship between perceived quality and loyalty intention for a service setting. Hypothesis 1 was thus supported.

**Model 2** assumes that satisfaction and perceived quality influence loyalty intention equivalently, as these two constructs are considered as one and the same. Previous research has shown a clearly discriminant validity between these two constructs. This indicates that perceived quality and customer satisfaction are distinct. As shown in Table 3, the regression coefficient of perceived quality–loyalty intention and customer satisfaction–loyalty intention constructs were different. The regression coefficient of perceived quality on loyalty intention (0.067, t = 0.761, p = 0.45) and customer satisfaction on loyalty intention (0.56, t = 6.18, p > 0.05) were unequal, but both were statistically significant. This result confirms that perceived quality and customer satisfaction are statistically distinct constructs, and that they influence customer loyalty intention differently. Thus, Hypothesis 2 was rejected.

**Model 3** tests the mediation role of perceived quality in the relationship between customer satisfaction and customer loyalty intention. As indicated in Table 3, the structural model for model 3 produced acceptable fit measures (chi-square 34.90 with 25 degrees of freedom; GFI = 0.949; SRMR = 0.035; RMSEA = 0.052). Model 3 supported the same regression coefficient as model 1 for the perceived quality, satisfaction and loyalty intention constructs. It indicated the effect of perceived quality on satisfaction (0.63, t = 13.38, p < 0.05), satisfaction on loyalty intention (0.48, t = 11.07, p < 0.05) and perceived quality on loyalty intention (0.262, t = 2.95, p < 0.05).

However, contrary to model 1, the role of perceived quality mediating the relationship between customer satisfaction and customer loyalty intention was not supported in model 3. The path coefficient of customer satisfaction to loyalty intention was not affected when perceived quality was introduced into the mediation model. Data results showed that the path coefficient of customer satisfaction to customer loyalty remained the same and continued at a statistically significant level (0.48, t = 11.07, p < 0.05). Meanwhile, the perceived quality to customer loyalty construct reached a non-statistically significant level (0.11, t = 1.16, p = 0.245). In conclusion, these results strongly suggest that the mediation role of perceived quality is nonexistent in the relationship between customer satisfaction and loyalty intention, and model 3 was thus rejected.

## Discussion

Because of strong competition in health services and changing consumer health attitudes, hospitals are now seeking to enhance patient loyalty using quality and satisfaction improvement strategies. If hospitals wish to be effective, it is critical that they clearly understand the relationship between these three concepts. In an effort to address this situation, this research empirically tested three theoretical models of perceived health service quality, patient satisfaction and patient loyalty relationship using a literature review as a basis for the studies. By integrating the mediation effect, this research also attempted to identify the role of mediation between these three constructs.

This study used hospital patients as participants. Empirical data came from a sample survey in six Chinese public hospitals. The investigated research models included three hypothesized relationships of these three constructs: 1) patient satisfaction mediates perceived quality and patient loyalty intention; 2) perceived quality and patient satisfaction have an equivalent impact on patient loyalty; and 3) perceived quality mediates patient satisfaction and patient loyalty intention. Construct reliability and validity were demonstrated by exploratory and confirmatory factor analysis for each construct. Relationships between each concept were analyzed using SEM.

### Model comparison

Our results confirm that in a health service encounter, perceived quality and patient satisfaction are distinct, and both constructs lead to patient loyalty intention. Through the path coefficient, a high and positive correlation between perceived quality and patient satisfaction was found. The correlation between quality–loyalty and satisfaction–loyalty were both positive, but weaker than the quality–satisfaction relationship.

Proposed theoretical model 1, with patient satisfaction as a mediator between healthcare perceived quality and patient loyalty, provides an acceptable fit for the model evaluation. It confirms that perceived quality and patient satisfaction both lead to patient loyalty intention, and that the relationship between quality and loyalty is largely influenced by patient satisfaction, because satisfaction acts as a mediator between quality–loyalty relationships. In this approach, the first model “patient satisfaction mediates the relationship between perceived quality and patient loyalty” was supported in our research.

Proposed theoretical model 2, with perceived quality and patient satisfaction both influencing patient loyalty equally, was not supported in this study. According to the model evaluation, the chi-square value was 89.03 with 26 degrees of freedom. This indicated that the model did not fit the data. Moreover, the path coefficients of quality–loyalty and satisfaction–loyalty were not equivalent. Thus, the second model was rejected.

Proposed theoretical model 3, with perceived quality as a mediator between the patient satisfaction and patient loyalty intention relationship, was not supported in this study. The path coefficient indicated that patient satisfaction impacts patient loyalty directly, and that perceived quality is not a mediator of the relationship between patient satisfaction and loyalty intention. Thus, model 3 was rejected.

### Limitations

Our study suffers from several limitations and we address these in combination with suggestions for future research avenues.

First, this study uses hospital inpatients as research participants. Compared with inpatients that generally spend a lot of time in one particular department, outpatients may have brief experiences with several departments, and thus be able to provide an overall evaluation of the service quality of that hospital. Therefore, the key dimensions of perceived quality may differ between inpatients and outpatients in a single hospital. According to Cho et al. [5], the single-site sample used in this study may represent a limitation to the generalizability of the findings.

Second, past studies on patient satisfaction have been geographically concentrated in the United States and Western Europe, with only a limited number addressing Asian countries (e.g., Korea, India and Malaysia). Few studies have focused on the mainland Chinese market (there have been previous studies on Hong Kong and Taiwan); thus, the findings of this study provide insight into the quality–satisfaction and loyalty relationship in the mainland Chinese market. The dimensionality and item content of perceived quality are different than those studies targeting U.S., European or Asian subjects. Thus, future studies could be conducted to compare the content validity of perceived quality instruments and cultural differences between healthcare consumers.

Third, the results presented here are based on analysis of a causal model with cross-sectional data. Research results support a priori causal effects. However, long-term effects cannot be inferred. Addressing this limitation represents an extensive exercise via longitudinal studies, and would be fruitful for future research.

Fourth, this study uses single-item measures of customer loyalty. Although recent results regarding the predictive validity of single items are good [45], the justification for their use in scientific marketing research continues to be debated [56-59].

Fifth, as expectations and perceived quality were measured at the same time, study results might suffer from a response shift problem [60]. A response shift problem might have occurred because adjustments could have occurred between expectations and perceived quality experiences.

Sixth, this research used SEM to compare the relationship between patient-perceived quality, patient satisfaction and loyalty intention using three models. For explanatory purposes, we did not focus on socio-demographic differences. This could also be a new direction for future research.

## Conclusion

This article supports the literature of Bitner [15]; Bolton and Drew [16]; Parasuraman, Zeithaml, and Berry [18,19]; Oliver [20]; Rust and Oliver [21]; and Zeithaml, Berry, and Parasuraman [22]. It emphasizes the distinction between perceived quality and customer satisfaction, with perceived quality as an antecedent of customer satisfaction in a service setting. From the empirical data, we confirm a mediation role of patient satisfaction in the relationship between perceived quality and patient loyalty. This result offers important implications for both marketing researchers and healthcare managers.

This study used the perceived service quality standard scale (SERVQUAL), adapted to suit health services. This scale was selected because of its widespread use. However, it focuses more on services provided than on the full spectrum of the patient experience [27]. Our adaptation of the SERVQUAL instrument using a single-dimensional measure confirms Babakus and Mangold’s [27] findings. Therefore, our results indicate that the adapted SERVQUAL scales can be used to assess service quality in the Chinese healthcare sector.

Other studies, however, have conceptualized service quality constructs using different numbers of dimensions. For instance, in the healthcare sector, Cho et al. [5] confirmed a four-dimensional structure of perceived health service quality in South Korea. Anbori et al. [61] identified a six-dimensional structure of perceived health service quality in Yemen. The nature of the concept of perceived health service quality is at the origin of this difference. In this paper, we use a reflective measure of the concept because we are interested in comparing models. For explanatory purposes, researchers might prefer formative models using causal links. The patient-reported experience concept [62] is formative by nature because it takes into account the various causes of perceived health service quality (e.g., doctor and nursing services, information examinations, organization, hospital and equipment). Such a focus is therefore multidimensional, and the indicators do not need to have strong co-variations [63].

## Competing interests

The authors declare that they have no competing interests.

## Authors’ contributions

JA designed the study. PL conducted the data collection. PL and JA analyzed the data. PL drafted the manuscript. JA and PL contributed to interpretation of findings and revision of the manuscript. JA supervised the study. All authors have read and approved the final manuscript.

## Pre-publication history

The pre-publication history for this paper can be accessed here:

http://www.biomedcentral.com/1472-6963/12/436/prepub
